# Genomic epidemiology of a densely sampled COVID-19 outbreak in China

**DOI:** 10.1093/ve/veaa102

**Published:** 2021-03-14

**Authors:** Lily Geidelberg, Olivia Boyd, David Jorgensen, Igor Siveroni, Fabrícia F Nascimento, Robert Johnson, Manon Ragonnet-Cronin, Han Fu, Haowei Wang, Xiaoyue Xi, Wei Chen, Dehui Liu, Yingying Chen, Mengmeng Tian, Wei Tan, Junjie Zai, Wanying Sun, Jiandong Li, Junhua Li, Erik M Volz, Xingguang Li, Qing Nie

**Affiliations:** 1 Department of Infectious Disease Epidemiology and MRC Centre for Global Infectious Disease Analysis, Imperial College London, Norfolk Place W2 1PG, UK; 2 Department of Mathematics, Imperial College London, London SW7 2AZ, UK; 3 Department of Microbiology, Weifang Center for Disease Control and Prevention, Weifang 261061, China; 4 Department of Respiratory Medicine, Weifang People’s Hospital, Weifang 261061, China; 5 Immunology Innovation Team, School of Medicine, Ningbo University, Ningbo 315211, China; 6 Shenzhen Key Laboratory of Unknown Pathogen Identification, BGI-Shenzhen, Shenzhen 518083, China; 7 Department of Hospital Office, The First People’s Hospital of Fangchenggang, Fangchenggang, 538021, China

**Keywords:** SARS-CoV-2, phylodynamics, phylogenetics, genetic epidemiology, structured coalescent, modelling

## Abstract

Analysis of genetic sequence data from the SARS-CoV-2 pandemic can provide insights into epidemic origins, worldwide dispersal, and epidemiological history. With few exceptions, genomic epidemiological analysis has focused on geographically distributed data sets with few isolates in any given location. Here, we report an analysis of 20 whole SARS- CoV-2 genomes from a single relatively small and geographically constrained outbreak in Weifang, People’s Republic of China. Using Bayesian model-based phylodynamic methods, we estimate a mean basic reproduction number (*R*_0_) of 3.4 (95% highest posterior density interval: 2.1–5.2) in Weifang, and a mean effective reproduction number (*R_t)_* that falls below 1 on 4 February. We further estimate the number of infections through time and compare these estimates to confirmed diagnoses by the Weifang Centers for Disease Control. We find that these estimates are consistent with reported cases and there is unlikely to be a large undiagnosed burden of infection over the period we studied.

## 1. Introduction

We report a genomic epidemiological analysis of one of the first geographically concentrated community transmission samples of SARS-CoV-2 genetic sequences collected outside of the initial outbreak in Wuhan, China. These data comprise 20 whole-genome sequences from confirmed COVID-19 cases in Weifang, Shandong Province, People’s Republic of China. The data were collected over the course of several weeks up to 10 February 2020, and overlap with a period of intensifying public health and social distancing measures. These interventions included public health messaging, establishing phone hot-lines, encouraging home isolation for recent visitors from Wuhan (January 23–26), optimising triage of suspected cases in hospitals (January 24), travel restrictions (January 26), extending school closures, and establishing ‘fever clinics’ for consultation and diagnosis (January 27) (Mao 2020). In contrast to the early spread of COVID-19 in Hubei Province of China, most community transmissions within Weifang took place after these measures were put in place.

Model-based phylodynamic methods have been previously used to analyse sequence data from Wuhan and exported international cases (Volz et al. 2020). Using an adaptation of these methods, and based on the local genetic data available, the objective of this study is to evaluate the growth rate and reproduction number in Weifang after seeding events that took place in mid to late January, 2020. A secondary aim is to provide estimates of the epidemiological trajectory of the Weifang outbreak and comparing them to confirmed diagnosed COVID-19 cases reported by Weifang Centers for Disease Control (CDC), to explore whether there was a significant unmeasured burden of infection due to imperfect case ascertainment from mild or asymptomatic illness.

## 2. Methods and materials

### 2.1 Epidemiological investigation, sampling and genetic sequencing

As of 10 February 2020, 136 suspected cases and 214 close contacts were diagnosed by Weifang Center for Disease Control and Prevention; of these, 38 cases were confirmed positive with SARS-CoV-2. The median age of patients was 36 (range: 6–75). Two of twenty patients suffered severe or critical illness.

Viral RNA was extracted using the Maxwell 16 Viral Total Nucleic Acid Purification Kit (Promega AS1150) with the magnetic bead method, and the RNeasy Mini Kit (QIAGEN 74104) with the column method. Quantitative reverse transcription polymerase chain reaction (RT-qPCR) was carried out using the 2019 novel coronavirus nucleic acid detection kit (BioGerm, Shanghai, China) to confirm the presence of SARS-CoV-2 viral RNA with cycle threshold (Ct) values ranging from 17 to 34, targeting the highly conservative region (ORF1ab/N gene) in the SARS-CoV-2 genome.

Concentration of RNA samples was measured by the Qubit RNA HS Assay Kit (Thermo Fisher Scientific, Waltham, MA, USA). The enzyme DNase was used to remove host DNA. The remaining RNA was used to construct the single-stranded circular DNA library with the MGIEasy RNA Library preparation reagent set (MGI, Shenzhen, China). Purified RNA was then fragmented. Using these short fragments as templates, random hexamers were used to synthesise the first-strand cDNA and then the second strand. Using the short double-strand DNA, a DNA library was constructed through end repair, adaptor ligation and PCR amplification. PCR products were transformed into a single-strand circular DNA library through DNA-denaturation and circularisation. DNA nanoballs (DNBs) were generated with the single-strand circular DNA library by rolling circle replication. The DNBs were loaded into the flow cell and pair-end 100 bp sequencing was performed on DNBSEQ-T7 platform 8 (MGI, Shenzhen, China). Twenty genomes were assembled with length from 26,840 to 29,882 nucleotides.

Total reads were first processed using Kraken v0.10.5 (default parameters) with a self-built database of Coronaviridae genomes (including SARS, MERS, and SARS-CoV-2 genome sequences downloaded from GISAID, NCBI, and CNGB) to identify Coronaviridae-like reads. To remove low-quality reads, duplications and adaptor contaminations, fastp v0.19.5 (parameters: -q 20-u 20 -n 1 -l 50) and SOAPnuke v1.5.6 (parameters: -l 20 -q 0.2 -E 50 -n 0.02 -5 0 -Q 2 -G -d) were used. The Coronaviridae-like reads of samples with <100× average sequencing depth were directly assembled de novo with SPAdes v3.14.0 using default settings. The Coronaviridae-like reads of samples with *>*100× average sequencing depth across the SARS-CoV-2 genome were subsampled to achieve 100× sequencing depth before being assembled.

The 20 Weifang sequences have mean 1.1 per cent N content and are deposited in GISAID (gisaid.org).

### 2.2 Mathematical model

The phylodynamic model is designed to account for 1, nonlinear epidemic dynamics in Weifang with a realistic course of infection (incubation and infectious periods), 2, variance in transmission rates that can influence epidemic size estimates, and 3, migration of lineages in and out of Weifang.

#### 2.2.1 Nonlinear epidemiological dynamics in Weifang

The maximum number of daily confirmed COVID-19 cases occurred on February 5, but it is unknown when the maximum prevalence of infection occurred. To capture a nonlinear decrease in cases following epidemic peak, and to account for a realistic distribution of generation times, we use an extension of the susceptible- exposed-infectious-recovered (SEIR) model (Keeling and Rohani 2011) for epidemic dynamics in Weifang, shown in Equations (1–5).

#### 2.2.2 Variance in transmission rates

To estimate total numbers infected, the phylodynamic model must account for epidemiological variables which are known to significantly influence genetic diversity (Lloyd-Smith et al. 2005). Foremost among these is the variance in offspring distribution (number of transmissions per primary case). We draw on previous evidence based on the previous SARS epidemic, which indicates that the offspring distribution is highly over-dispersed. High variance of transmission rates will reduce genetic diversity of a sample and failure to account for this factor will lead to highly biased estimates of epidemic size (Li et al. 2017). Recent analyses of sequence data drawn primarily from Wuhan have found that high over-dispersion was required for estimated cases to be consistent with the epidemiological record (Volz et al. 2020). Models assuming low variance in transmission rates between people would generate estimates of cases that are lower than the known number of confirmed cases. Separately, Endo (2020) found that high over-dispersion is required to reconcile estimated reproduction numbers with the observed frequency of international outbreaks. We therefore elaborate the SEIR model with an additional compartment *J* which has a higher transmission rate (*τ* -fold higher) than the *I* compartment.

The variance of the implied offspring distribution is calibrated to give a similar over-dispersion to that of the SARS epidemic. Upon leaving the incubation period, individuals progress to the *J* compartment with probability *p_h,_* or otherwise to *I*. The model is implemented as a system of ordinary differential equations:

1 $$\dot{S}\left(t\right)=-(\beta I\left(t\right)+\beta \tau J\left(t\right))\frac{S(t)}{S\left(t\right)+E\left(t\right)+I\left(t\right)+J\left(t\right)+R(t)}$$

2 $$\dot{E}\left(t\right)=\left(\beta I\left(t\right)+\beta \tau J\left(t\right)\right)\frac{S\left(t\right)}{S\left(t\right)+E\left(t\right)+I\left(t\right)+J\left(t\right)+R\left(t\right)}\gamma_{0}E(t)$$

3 $$\dot{I}\left(t\right)=\gamma_{0}\left(1-p_{h}\right)E\left(t\right)-\gamma_{1}I(t)$$

4 $$\dot{J}\left(t\right)=\gamma_{0}p_{h}E\left(t\right)-\gamma_{1}J(t)$$

5 $$\dot{R}\left(t\right)=\gamma_{1}\left(I\left(t\right)+J\left(t\right)\right)$$

#### 2.2.3 Importation of lineages from Wuhan

The outbreak in Weifang was seeded by multiple lineages imported at various times from the rest of China. We therefore account for location of sampling in our model. Migration is modelled as a bi-directional process with rates proportional to epidemic size in Weifang. The larger reservoir of COVID-19 cases outside of Weifang (*Y* (*t*)) serves as a source of new infections and is assumed to be growing exponentially (at rate *ρ*) over this time period.

The equation governing this population is:

6 $$\dot{Y}\left(t\right)=\left(\rho -\mu \right)Y(t)$$

Migration only depends on the size of variables in the Weifang compartment and thus does not influence epidemic dynamics; it will only influence the inferred probability that a lineage resides within Weifang. For compartment *X* (*E*, *I*, or *J)* , *η* is the per-lineage rate of migration out of Weifang, and the total rate of migration in and out of Weifang is *ηX*.

#### 2.2.4 Model fitting

During phylodynamic model fitting *η*, *β* and *ρ* are estimated. Additionally, we estimate initial sizes of *Y, E*, and *S*. Initial values of *I, J*, and *R* are fixed at 0. Other parameters are fixed based on prior information. We fix 1*/γ*_0_ = 4.1 days and 1*/γ*_1_ = 3.8 days (Volz et al. 2020). We set *p_h_* = 0.20 and *τ  *=  74 which yields a dispersion of the reproduction number that matches a negative binomial distribution with *k *=* *0.124 for any value of *R*_0_ between 2 and 5. This dispersion is similar to values estimated for the 2003 SARS epidemic (Lloyd-Smith et al. 2005).

**Figure 1.**
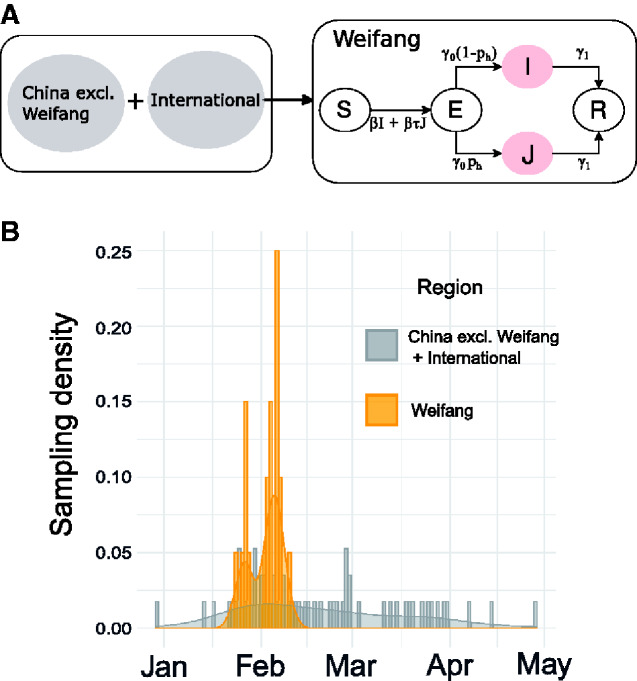
Epidemiological model and sample times. (A) A diagram representing the structure of the epidemiological SEIR model which was fitted in tandem with the time scaled phylogeny. Infected and infectious individuals may occupy a low (I) or a high (J) transmission rate state to account for high dispersion of the reproduction number. (B) Sampling density of sequences from inside (yellow) and outside (grey) of Weifang respectively through time.

The phylodynamic model is illustrated in Fig. 1A as a flowchart. The SEIR model dynamics begin on 10 January.

It is important to note that the *S* compartment does not explicitly represent the number of susceptibles in Weifang, but rather it is used as a simple parameterisation to permit *R_t_* to decrease, and for epidemic control to be achieved. An exploration of this parameterisation is discussed in Supplementary Information Section 4.1.

### 3. Phylogenetic analysis

Using, MAFFT (Katoh and Standley 2013), we aligned the 20 Weifang sequences with a previous alignment of 57 non-identical SARS-CoV 2 sequences from outside of Weifang, hereby the ’reference set’ (Volz et al. 2020). The reference set was sampled from the GISAID database (Elbe and Buckland-Merrett 2017) downloaded on June 7, 2020, and explicitly included close genetic matches to sequences from Weifang. An upper bound at 1 May on the date of sampling was placed. The distribution of sample dates from inside and outside of Weifang is shown in Fig. 1B. Of the 57 sequences in the reference set, 20 (35%) were sampled from China.

Maximum likelihood analysis was carried using IQTree (Minh et al. 2019) with a HKY+G4 substitution model, and a time-scaled tree was estimated using treedater 0.5.0 (Volz and Frost 2017).

Bayesian phylogenetic analysis was carried out using BEAST 2.6.1 (Bouckaert et al. 2019) with a HKY+G4 substitution model and a strict molecular clock. The phylodynamic model was implemented using the PhyDyn package v1.3.7 (Volz and Siveroni 2018) using the QL likelihood approximation and the RK ODE solver. The model was fitted by running 8 MCMC chains of 30 million steps in parallel, and combining chains after removing 50 per cent burn-in. In order to demonstrate the added utility of the sequence data, the analysis was repeated assuming a constant likelihood, that is sampling only from the prior probability distributions.

The *ggtree* package was used for all phylogeny visualisations (Yu et al. 2017).

Code to replicate this analysis and BEAST XML files can be found at https://github.com/emvolz/weifang-sarscov2.

## 4. Results

Despite an initial rapid increase in confirmed cases in Weifang in late January and early February, the number of confirmed cases by Weifang CDC show that the outbreak peaked early and the maximum number of cases occurred on 5 February. Phylodynamic analysis supports the interpretation that control efforts reduced epidemic growth rates and contributed to eventual control. Estimates of the epidemiological parameters are summarised in Table 1.

**Table 1.** Summary of primary epidemiological and evolutionary parameters, including Bayesian prior distributions and estimated posteriors.

Parameter	Prior	Posterior mean	95% HPD
Initial infected	Exponential (mean = 1)	4.8	1.3–10.1
Initial susceptible	Exponential (mean = 500)	550	117–1501
Migration rate^a^	Exponential (mean = 10)	1.68	1.03–1.99
Transmission rate	Log-normal (mean log = 3.21, SD log = 0.5)	21.5	13.0–32.1
Reproduction number	Log-normal (mean log = 1.03, SD log = 0.5)	3.4	2.1–5.2
Molecular clock rate^b^	Uniform (0.0007,0.003)	0.0013	0.00098–0.0017
Transition/transversion	Log-normal (mean log = 1, SD log = 1.25)	4.6	3.3–6.5
Gamma shape	Exponential (mean = 1)	0.29	0.0070–1.50

Posterior uncertainty is summarised using a 95 per cent HPD interval.

a Units: Migrations per lineage per year.

b Units: Substitutions per site per year.

**Figure 2.**
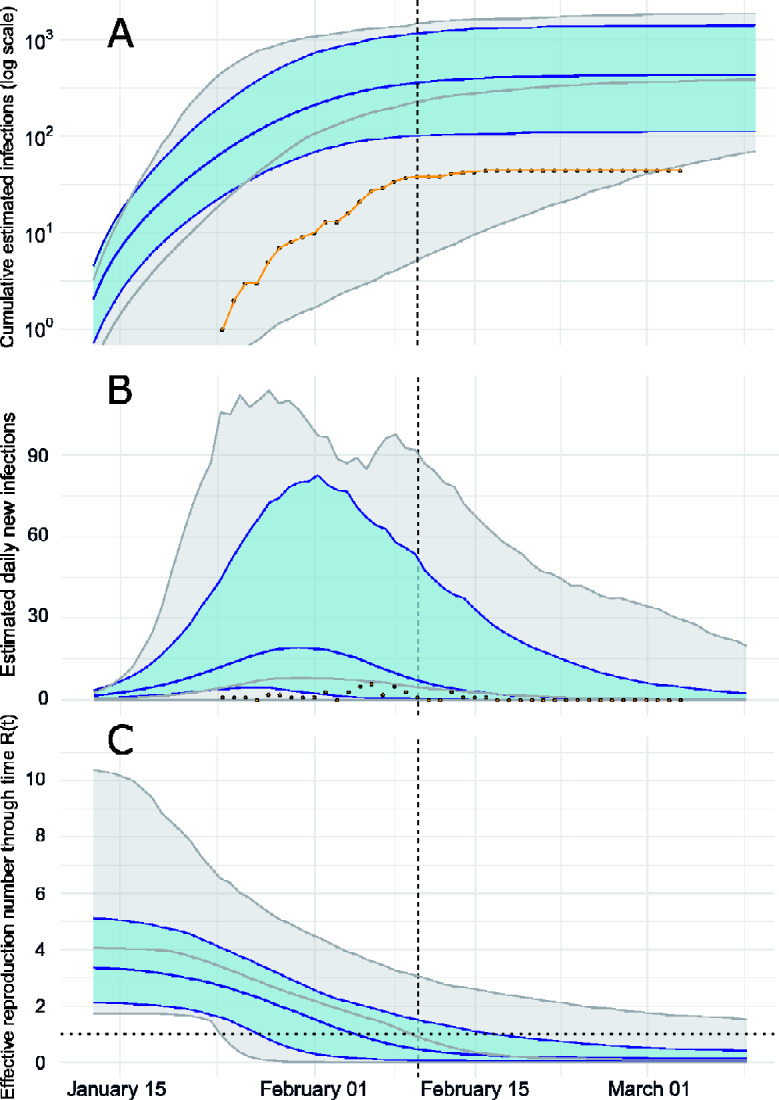
Epidemiological trajectory of the Weifang SARS-CoV-2 epidemic in 2020 when fitting the SEIR model to genetic data (blue) and sampling only from prior (grey). Solid lines and shaded area reflect posterior median and 95 per cent HPD. The vertical dashed line represents the date of the last sequence sampled in Weifang. (A) Cumulative estimated infections through time compared with cumulative cases (yellow points) reported by Weifang CDC. (B) Daily estimated infections through time compared to daily reported cases (yellow points). (C) Effective reproduction number through time *R_t_*. The horizontal dotted line indicates *R_t_* = 1.

The estimated cumulative and daily number of infections are shown in Fig. 2A and B, respectively. We estimate the peak of daily infections in late January, preceding the time series of confirmed cases by about a week; this is expected due to delays from infection to appearance of symptoms and delays from symptoms to diagnosis. The genetic data are strongly informative about timing and size of the epidemic peak: trajectories sampled from the Bayesian prior distribution have a smaller and later epidemic peak (c.f. Fig. 2) with much less precision. Our central estimate for the cumulative number infected on 10 February is 365 (highest posterior density (HPD) 102–1174), compared with 38 cumulative confirmed cases. We therefore estimate that around 10 per cent of infections were diagnosed (Supplementary Fig. S5), an unknown proportion of infections will be missed by the surveillance system due to very mild, sub-clinical or asymptomatic infection. This supports the hypothesis that there was a modest (but not large) burden of infection in Weifang over the period that the sequence data were sampled.

Effective reproduction number over time is shown in Fig. 2C. We estimate *R*_0_ = 3.4 (95% HPD: 2.1–5.2) and the initial growth rate in cases was approximately 22 per cent per day, consistent with those estimated in other settings and during the early epidemic in Wuhan (Alimohamadi et al. 2020). Sampling from the prior yields a much higher estimate for *R*_0_ with an unrealistic HPD upper bound over 10. We detect a significant decrease in effective reproduction number as the epidemic progressed, during a period (late January) when Weifang was implementing a variety of public health interventions and contact tracing to limit epidemic spread. Our central estimate of *R_t_* drops below 1 on the 4th of February.

Although previous studies have shown the significance of realistic modelling for fidelity of phylogenetic inference (M¨oller et al. 2018), our analysis has found that the phylodynamic prior did not greatly influence estimated molecular clock rate or inferred time to most recent common ancestors (TMRCAs). This is likely due to our choice of reference sequence set, which comprised sequences spanning several months of the epidemic, and therefore reflecting a range of transmission dynamics.

In this analysis, there is a mean of three pairwise differences among sequences from Weifang; the corresponding number among the sequences outside of Weifang is eight.

**Figure 3.**
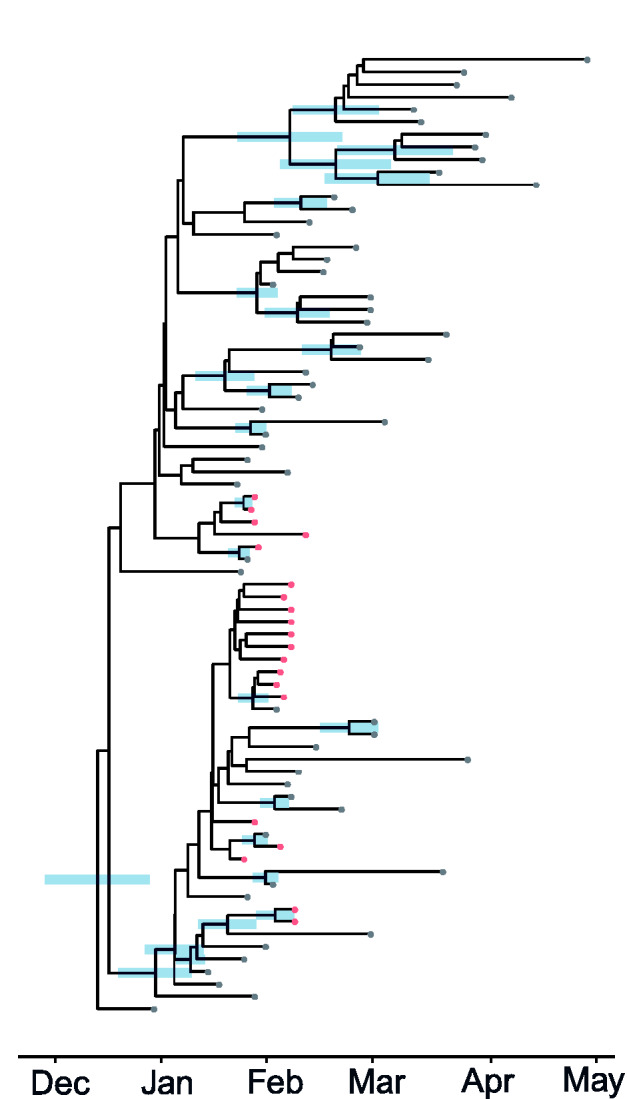
A time-scaled phylogeny (MCC tree) co-estimated with epidemiological parameters of the SARS-CoV-2 epidemic in 2020. Red and grey tips correspond to samples from inside and outside Weifang, China, respectively. The credible interval of time to most recent common ancestor (TMRCA) is shown as a blue bar for all nodes with more than 50 per cent posterior probability support.

Figure 3 shows the estimated time-scaled maximum clade credibility (MCC) tree including 20 lineages sampled from distinct patients in Weifang and 57 genomes sampled from Wuhan and internationally.

There is correspondingly low confidence in tree topology (Supplementary Fig. S3), and only two monophyletic Weifang clades had greater than 50 per cent posterior probability, neither of which are larger than two samples.

The earliest Weifang sequence was sampled on 25 January from a patient who first showed symptoms on 16 January. These dates cover a similar range as the posterior TMRCA of all Weifang sequences (Supplementary Fig. S4).

## 5. Discussion

Our analysis of 20 SARS-CoV-2 genomes has confirmed independent observations regarding the rate of spread and burden of infection in Weifang, China. Surveillance of COVID-19 is rendered difficult by high proportions of illness with mild severity and an unknown proportion of asymptomatic infection (Guan 2019). The extent of under-reporting and case ascertainment rates has been widely debated. Analysis of genetic sequence data provides an alternative source of information about epidemic size. We do not find evidence for a large hidden burden of infection within Weifang, with an estimated total number of cases around 365 (102–1174) at the date of last sample, towards the end of the outbreak.

Our decreasing central estimate of *R_t_* over time, falling below 1 on 4 February, suggests a slower rate of spread outside of Wuhan and effective control strategies implemented in late January. It is consistent with a previous modelling study of Shandong province (Zhang et al. 2020), which showed that *R_t_* fell below 1 on 29 January. Our posterior molecular clock rate shown in Table 1 is consistent with previous estimates of SARS-CoV-2 phylogenetic analyses (Nie et al. 2020).

The modest number of sequences from Weifang (twenty) is a limitation of this study. However, this represents a significant proportion of the total number of cases reported; there were thirty-eight confirmed cases at the date of the last genetic sample (10 February), rising no further than forty-four from 16 February onwards (Fig. 2A). Despite relatively few sequences, our estimated trajectories display uncertainties that are significantly reduced and more realistic, compared with sampling only from the prior.

Further, it is possible that the outbreak observed in Weifang could be due not to community transmission, but rather multiple importations. However, given that we sampled the reference set from a GISAID database downloaded in June, it is reasonable to assume close genetic matches would have been chosen. A maximum-likelihood tree of the entire alignment (Supplementary Fig. S1) shows that lineages from Weifang have common ancestry with other Chinese lineages at two distinct polytomies and the phylogeny alone gives no information about location of these nodes (Weifang or exogenous). We therefore conclude that the MCC in Fig. 3, which reflects significant clustering of the Weifang samples, is reasonable.

Community transmission is further supported by the fact that cases were identified via contact tracing. This forms another limitation, as it suggests non-random sampling of cases in Weifang. This could lead to an underestimate of the total number of cases in Weifang. However, as a large proportion of reported cases were included in this analysis, the bias is unlikely to be too significant.

Finally, the SEIR model structure also presents some limitations. As *β* has a constant value, *R_t_* can decrease only as a result of depleting susceptibles. The decrease in *R_t_* is therefore a constraint in the model and occurred even when sampling from the prior. Despite this, the genetic data was informative on the value of *β* (and therefore *R*_0_), which in turn affects the date at which *R_t_* falls below 1. Our analysis demonstrates a reliable mean estimate of *R*_0,_ with a narrower uncertainty, compared to sampling from the prior. Although other methods which allow for time-varying transmission rate (including other PhyDyn model templates) or models with a piece-wise *R_t_* function (Frost and Volz 2010), our SEIR-type model with constant *β* required fewer parameters, appropriate for an analysis with only 20 internal sequences.

While the value of pathogen genomic analysis is widely recognised for estimating dates of emergence (Gire et al. 2014) and identifying animal reservoirs (Dudas et al. 2018; Zhou et al. 2020), analysis of pathogen sequences also has the potential to inform epidemic surveillance and intervention efforts. This is demonstrated clearly in our analysis, drawing on previously developed models and packages for BEAST2 (Volz and Siveroni 2018; Bouckaert et al. 2019), where our results show much narrower uncertainties and more realistic estimates compared with sampling from the prior. Indeed, the added value of fitting to only 20 local sequences in this analysis demonstrates the utility of phylodynamic modelling for outbreaks as compared with traditional epidemiological modelling fitted only to case data.

We also demonstrate a pipeline for real-time phylodynamic analysis, which could feasibly provide realistic results as a supplement to epidemiological surveillance. The analysis described in this report was accomplished within 48 hours; however, the real-time utility of such methods is dependent on randomised concentrated sampling within localities, coupled with timely sharing of data. Efficient genetic sequencing, processing and data sharing, coupled with phylodynamic analysis, could prove to be a key tool in the outbreak response toolkit.

## Data availability

Genetic sequence data are available from GISAID (gisaid.org). Accession numbers for sequences from Weifang: EPI_ISL_413691 EPI_ISL_413693 EPI_ISL_413694, EPI_ISL_413695 EPI_ISL_413696 EPI_ISL_413697, EPI_ISL_413711 EPI_ISL_413729 EPI_ISL_413746, EPI_ISL_413747 EPI_ISL_413748 EPI_ISL_413749, EPI_ISL_413750 EPI_ISL_413751 EPI_ISL_413752, EPI_ISL_413753 EPI_ISL_413761 EPI_ISL_413791, EPI_ISL_413809 EPI_ISL_413692. Accession numbers for sequences from outside of Weifang: EPI_ISL_414380 EPI_ISL_437621 EPI_ISL_429092, EPI_ISL_418327 EPI_ISL_416335 EPI_ISL_413854, EPI_ISL_402121 EPI_ISL_408480 EPI_ISL_418503, EPI_ISL_450196 EPI_ISL_417030 EPI_ISL_424356, EPI_ISL_451351 EPI_ISL_408010 EPI_ISL_430742, EPI_ISL_416366 EPI_ISL_451343 EPI_ISL_416381, EPI_ISL_407988 EPI_ISL_413882 EPI_ISL_413881, EPI_ISL_413879 EPI_ISL_411954 EPI_ISL_417184, EPI_ISL_418992 EPI_ISL_454935 EPI_ISL_414569, EPI_ISL_416570 EPI_ISL_416600 EPI_ISL_413608, EPI_ISL_451347 EPI_ISL_419242 EPI_ISL_414485, EPI_ISL_414005 EPI_ISL_430847 EPI_ISL_415580, EPI_ISL_413595 EPI_ISL_455376 EPI_ISL_417101, EPI_ISL_417168 EPI_ISL_455410 EPI_ISL_424081, EPI_ISL_440461 EPI_ISL_440433 EPI_ISL_455696, EPI_ISL_444577 EPI_ISL_456208 EPI_ISL_434463, EPI_ISL_437264 EPI_ISL_452673 EPI_ISL_437515, EPI_ISL_437185 EPI_ISL_427257 EPI_ISL_432722, EPI_ISL_437704 EPI_ISL_461275 EPI_ISL_403932.

## Supplementary data

Supplementary data are available at *Virus Evolution* online.

## Supplementary Material

veaa102_Supplementary_Data
