# Supplementary material for: Genomic epidemiology of a densely sampled COVID-19 outbreak in China
Source: Virus Evol. 2021 Mar 14;7(1):veaa102. doi: 10.1093/ve/veaa102 (PMC7955981; doi:10.1093/ve/veaa102)
Supplement: veaa102_Supplementary_Data [file veaa102_supplementary_data.pdf]

Genomic epidemiology of a densely sampled COVID-19 outbreak in China: supplementary information

Lily Geidelberg[1*], Olivia Boyd[1], David Jorgensen[1], Igor Siveroni[1], Fabricia F. Nascimento[1], Robert Johnson[1], Manon Ragonnet-Cronin[1], Han Fu[1], Haowei Wang[1], Xiaoyue Xi[2], Wei Chen[3], Dehui Liu[3], Yingying Chen[3], Mengmeng Tian[3], Wei Tan[4], Junjie Zai[5], Wanying Sun[6], Jiandong Li[6], Junhua Li[6], Erik M Volz[1*], Xingguang Li[7], and Qing Nie[3]

[1]Department of Infectious Disease Epidemiology and MRC Centre for Global Infectious Disease Analysis, Imperial College London, Norfolk Place, W2 1PG, United Kingdom
[2]Department of Mathematics, Imperial College London, London SW7 2AZ, United Kingdom
[3]Department of Microbiology, Weifang Center for Disease Control and Prevention, Weifang 261061, China
[4]Department of Respiratory Medicine, Weifang People's Hospital, Weifang 261061, China
[5]Immunology Innovation Team, School of Medicine, Ningbo University, Ningbo 315211, China
[6]Shenzhen Key Laboratory of Unknown Pathogen Identification, BGI-Shenzhen, Shenzhen 518083, China
[7]Department of Hospital Office, The First People's Hospital of Fangchenggang, Fangchenggang, 538021, China
[*]Corresponding author: Lily Geidelberg, l.geidelberg@ic.ac.uk
[*]Corresponding author: Dr Erik Volz, e.volz@imperial.ac.uk

November 25, 2020

1 Phylogenetic analysis

Below are a series of plots describing a phylogenetic analysis of the alignment used in this study. This includes (1) a maximum likelihood tree, (2) a root-to-tip regression, (3) a tree density plot and (4) estimated time to most recent common ancestor (TMRCA) of Weifang lineages.

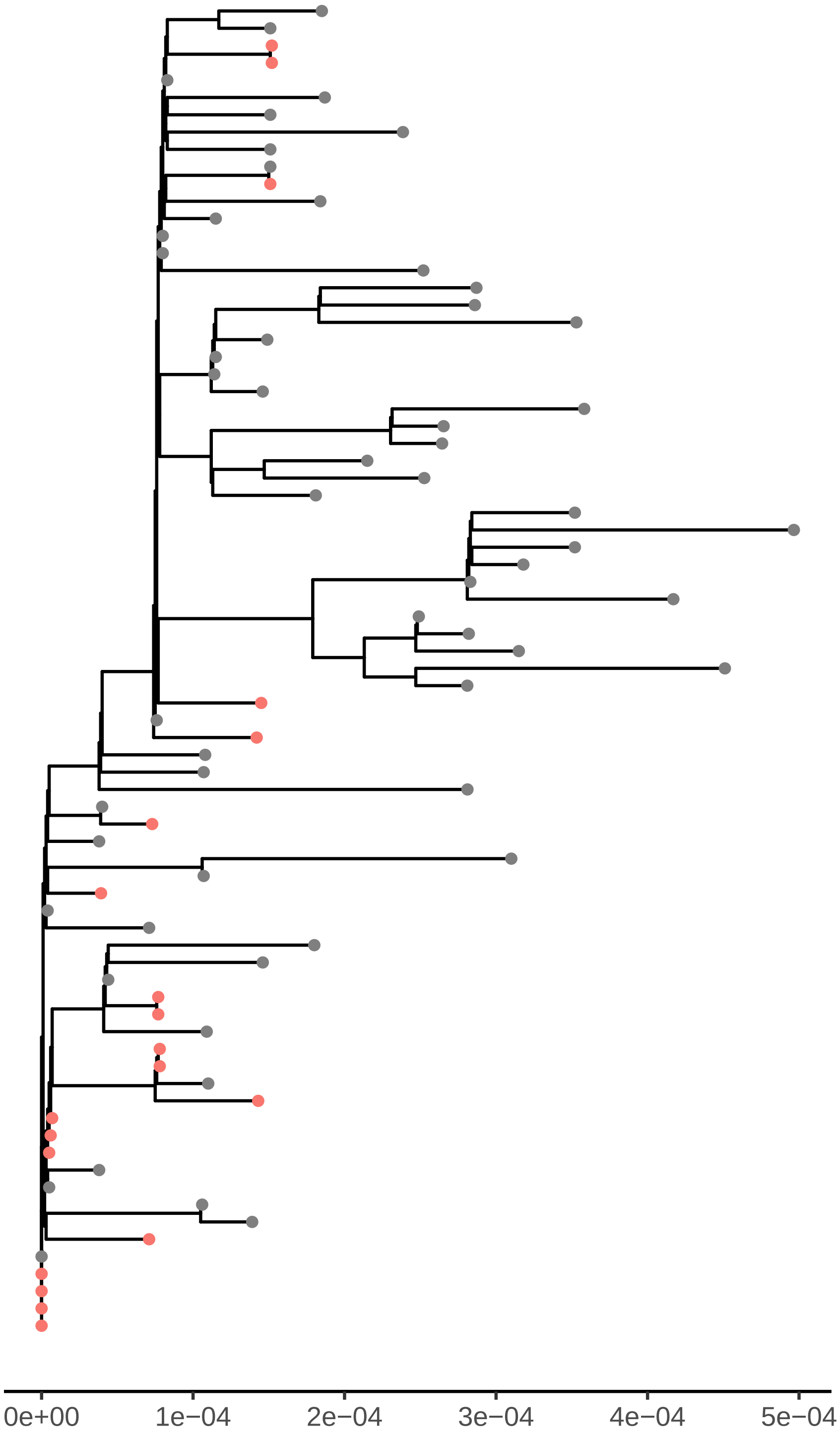

Figure S1: A maximum likelihood tree estimated using IQTree using the same data as used for the Bayesian analysis. Red and grey dots represent samples inside and outside Weifang respectively.

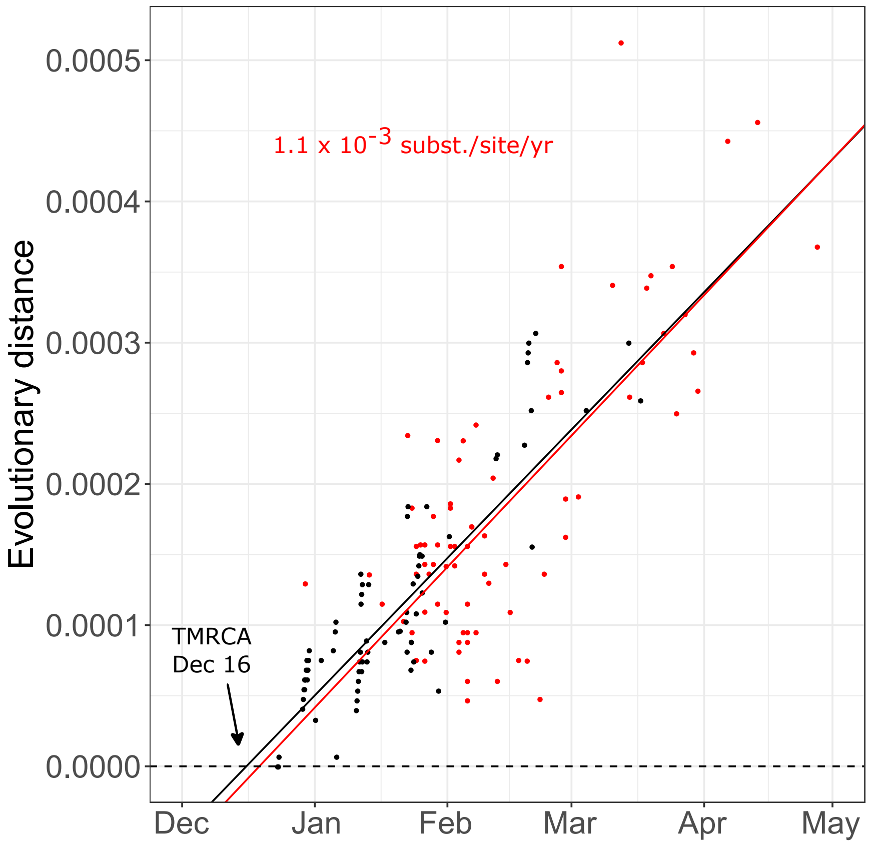

Figure S2: A root to tip regression (red and black points indicate sample and internal nodes respectively) showing approximately linear increase in diversity with time of sampling. The red text indicates the mean substitution rate.

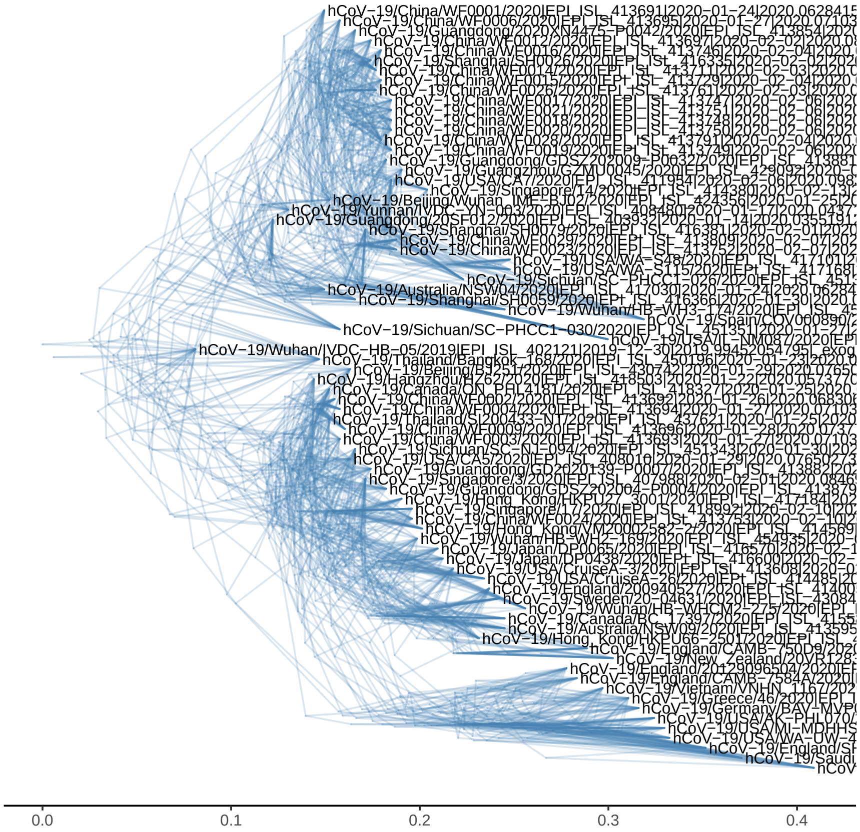

Figure S3: A tree density plot based on the posterior distribution of trees computed in BEAST2.

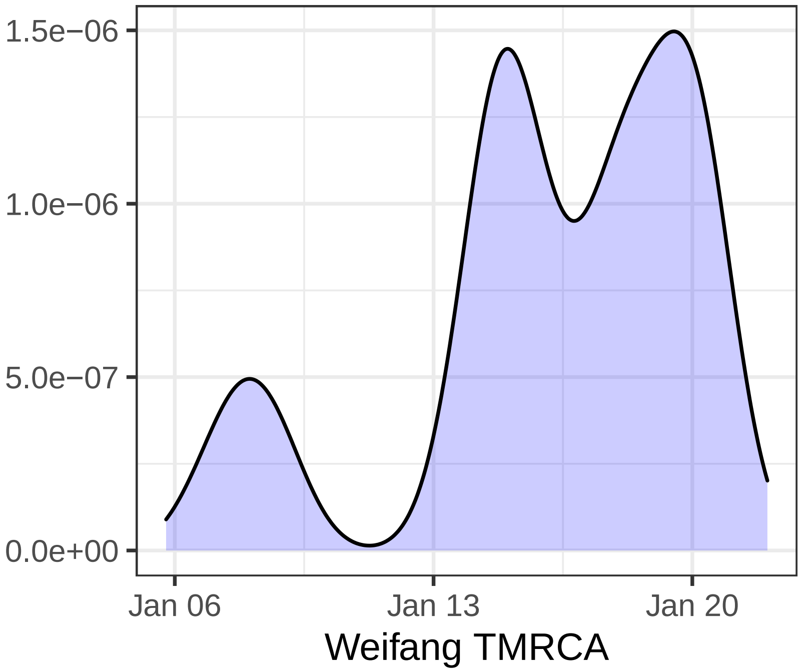

Figure S4: The estimated posterior TMRCA among all Weifang lineages.

2 Posterior parameters and effective sample sizes

Below are the mean values for the posterior parameter distributions of our phylodynamic analysis. We present here the results from the main analysis, with the parameterisation described in the main text. We also include the results from the same analysis when assuming a constant likelihood (sampling from the prior).

Table S1: Summary of primary parameters for the main analysis, including mean estimated posterior and effective sample size due to autocorrelation.

Statistic	Mean	ESS
Posterior	-41631.97352	1208
Likelihood	-41618.79558	1383
Prior	-13.17793938	1160
Tree likelihood	-41618.79558	1383
Tree height	0.371177175	2947
Molecular clock rate	0.00130656	1784
Gamma shape	0.286880065	2405
Transition/transversion	4.623272977	12172
PhydynSEIR	7.89341053	1130
Initial exposed	4.84479347	2956
Initial susceptible	549.8789703	3182
Transmission rate	21.45699354	4734
Initial exogenous	0.520109449	1486
Exogenous growth rate	20.47238551	1646
Migration rate	1.684142341	9309

Table S2: Summary of primary parameters for the main analysis but sampling from the prior, including mean estimated posterior and effective sample size due to autocorrelation.

Statistic	Mean	ESS
Posterior	-41643.01783	10410
Likelihood	-41626.68793	10272
Prior	-16.3299023	18874
Tree likelihood	-41626.68793	10272
Tree height	0.604156711	13422
Molecular clock rate	0.000709584	22985
Gamma shape	0.297625411	3313
Transition/transversion	4.62191852	13931
PhydynSEIR	0	NA
Initial exposed	1.001445038	23576
Initial susceptible	596.9800995	24008
Transmission rate	28.46598941	23490
Initial exogenous	1.00446398	24008
Exogenous growth rate	37.79539512	23614
Migration rate	0.968908965	8496

3 Reporting frequency

Below is the estimated reporting frequency of COVID-19 cases in Weifang, calculated by dividing the number of reported cases by the cumulative mean (and 95% HPD) estimated cases.

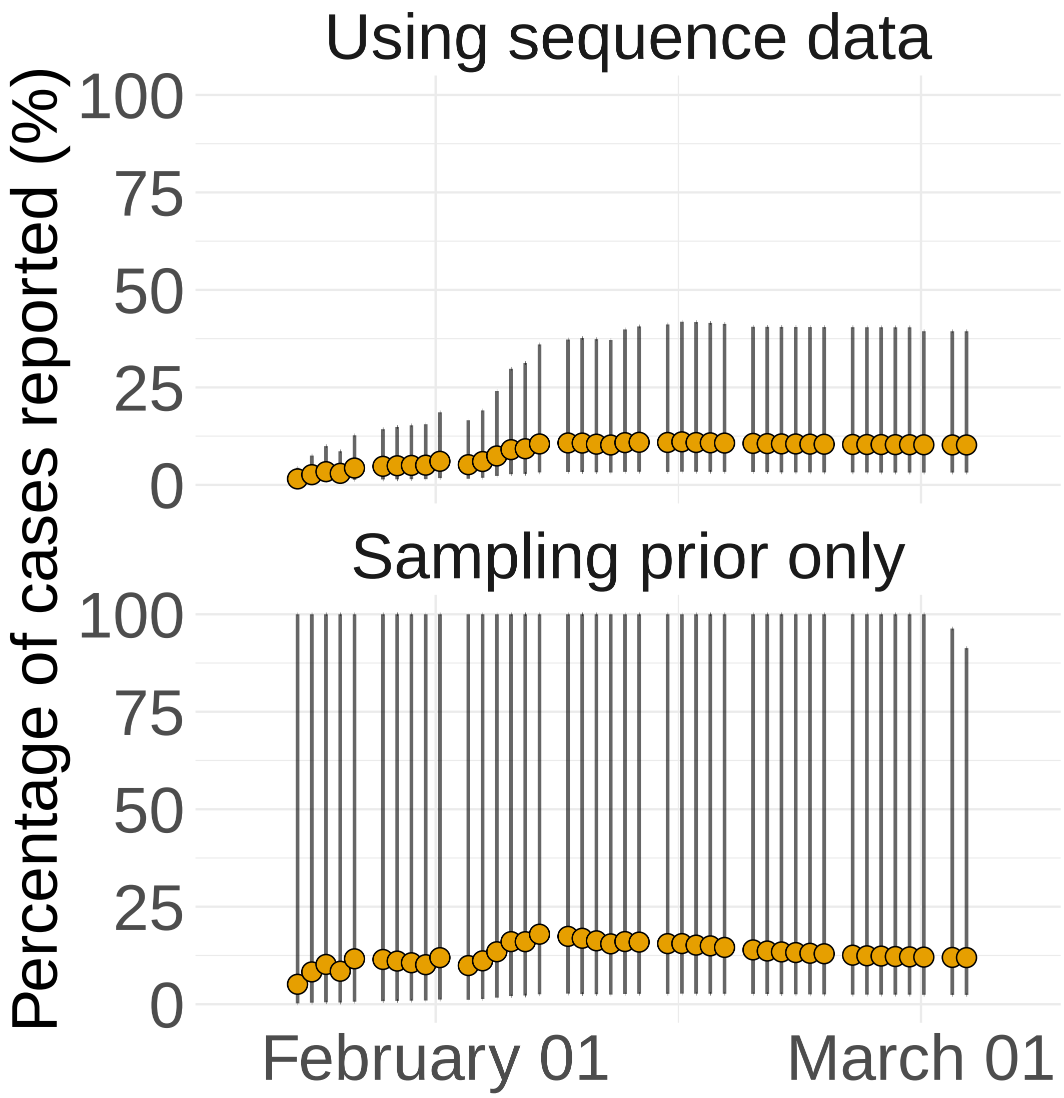

Figure S5: Estimated reporting frequency. The yellow points and grey bars reflect the mean and 95% HPD cumulative estimated proportion of cases that were identified through time respectively. Results shown separately for analyses conducted with and without the sequence data.

4 Sensitivity analyses

4.1 Initial number of susceptibles

In our analysis, we assumed a prior mean of 500 for the initial susceptible compartment. We performed a sensitivity analysis on this parameter, changing it from S=500 to S=9,086,241, the latter reflecting the total population of Weifang. Figure S6 shows that the reported number of cases is within the HPD of the estimated cumulative infections, and slightly below the central estimate. However, the estimated reproduction number remains constant above 1. We have strong prior belief that the outbreak was controlled (and therefore that R_t must have fallen below 1), and therefore believe these to be unrealistic posterior trajectories, highlighting the impossibility of such a high S prior.

It is important to note that the "susceptibles" compartment should be interpreted phenomenologically rather than mechanistically. We use it as a simple parameterisation for R_t to decrease, and for epidemic control to be achieved. We are not explicitly estimating the number of susceptibles in Weifang; this sensitivity analysis demonstrates that the potential for epidemic decline in our model framework (within a sensible timeframe) is dependent on certain model parameterisations, i.e. a small S prior.

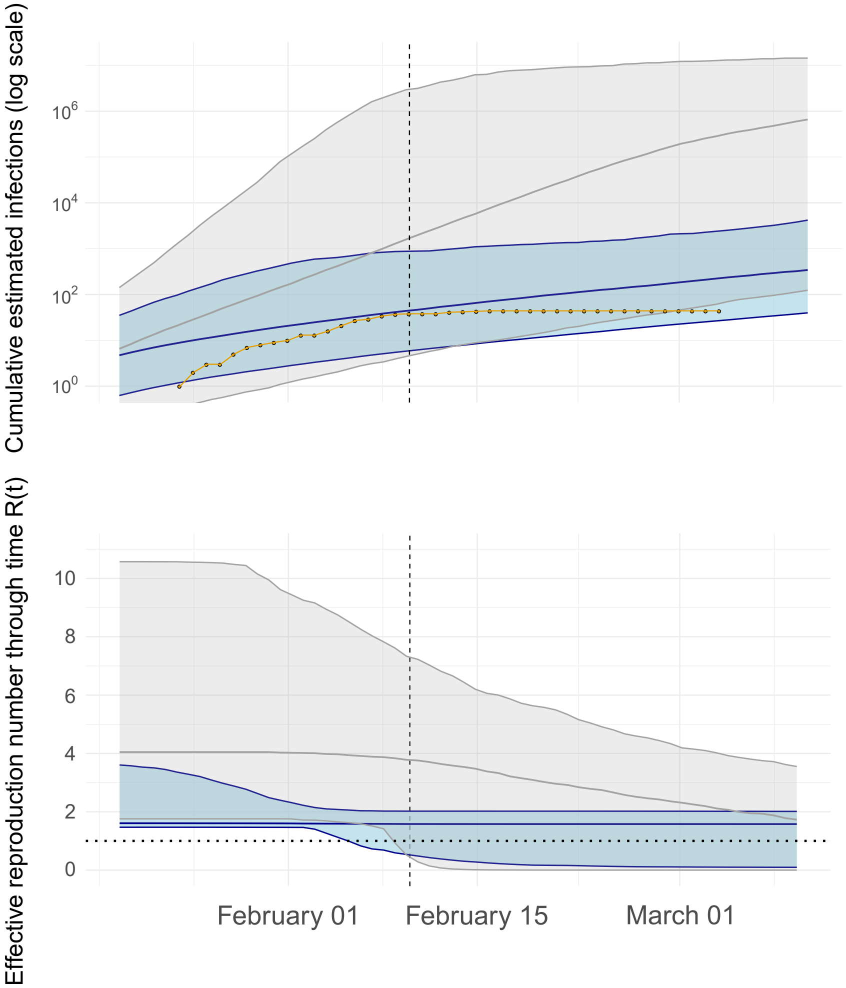

Figure S6: Assuming a mean initial susceptible prior of S = 9,086,241, cumulative estimated infections and effective reproduction number through time are shown when fitting the SEIR model to genetic data (blue) and sampling only from prior (grey). Solid lines and shaded area reflect posterior median and 95% HPD. The vertical dashed line represents the date of the last sequence sampled in Weifang; the horizontal dashed line represents $R(t) = 1$. Cumulative cases (yellow points) reported by Weifang CDC.

4.2 Offspring distribution heterogeneity

We performed another sensitivity analysis on the heterogeneity of offspring distribution. In the main analysis, the prior distribution of R_0 has mean around 4, which matched a negative binomial distribution with $k = 0.124$, similar to previous modelling from Endo et al 2020 (Endo *et al.*, 2020). It is worth noting that in our model, k is largely insensitive to R_0, and very sensitive to τ and p_h. We repeated our analysis while exploring lower offspring heterogeneity by assuming $\tau = 13$, which resulted in increasing k to 0.2. To maintain a similar mean prior R_0 of around 4 (as R_0 is very sensitive to τ), we increased the prior log-normal distribution mean of beta to 4.61 (sd=0.5). Under this parameterisation, the estimated number of infections at last sample (February 10) is 300 (49-1138), compared to 365 (102-1174) in the main analysis. The HPDs are highly overlapping and the conclusions remain the same, but the analysis with lower offspring heterogeneity, as expected, has fewer estimated cases.

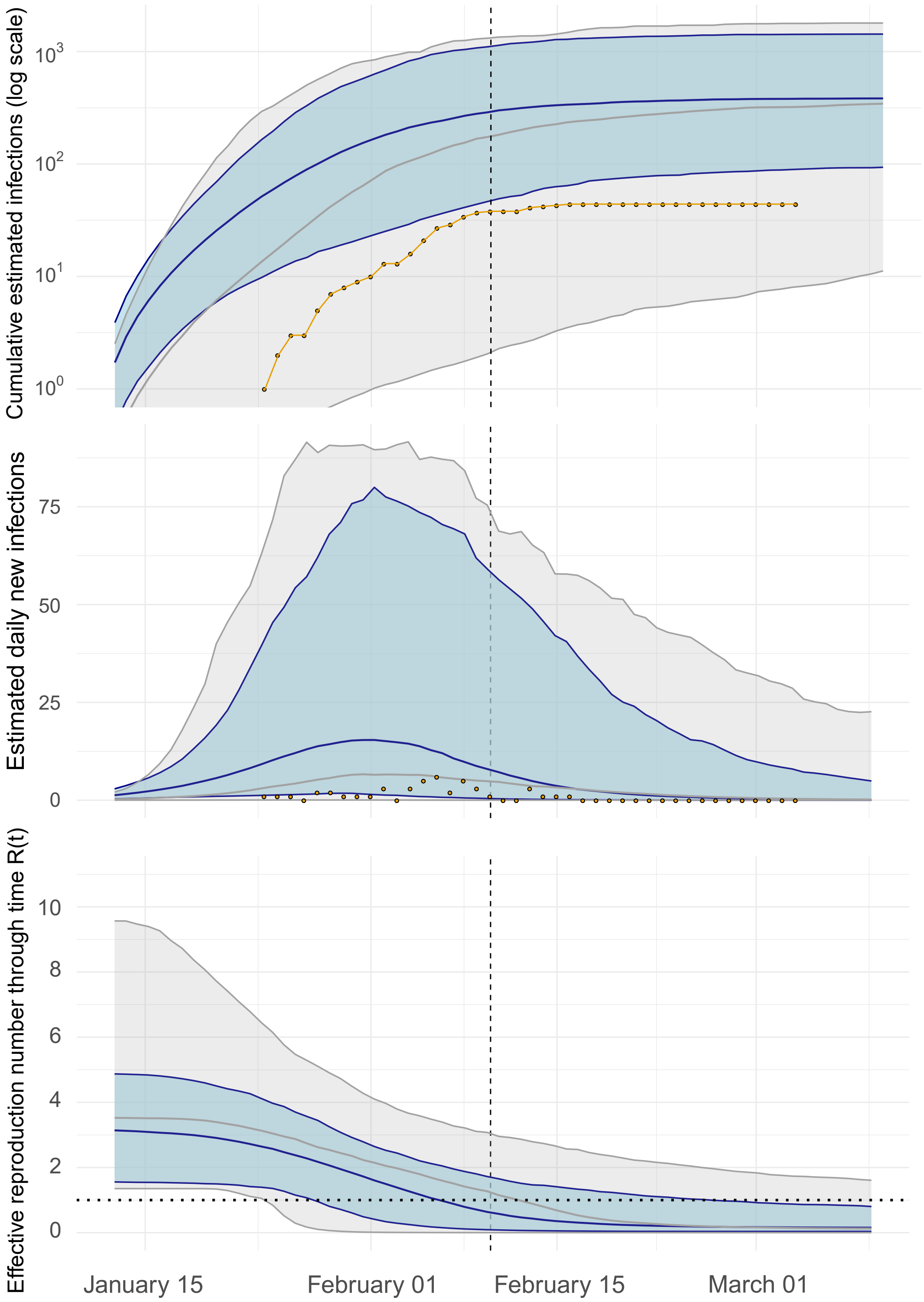

Figure S7: Assuming $\tau = 13$ (corresponding to dispersion $k = 0.2$), cumulative and daily estimated infections and effective reproduction number through time are shown when fitting the SEIR model to genetic data (blue) and sampling only from prior (grey). Solid lines and shaded area reflect posterior median and 95% HPD. The vertical dashed line represents the date of the last sequence sampled in Weifang; the horizontal dashed line represents $R(t) = 1$. Cumulative cases (yellow points) reported by Weifang CDC.

References

Endo, A., Abbott, S., Kucharski, A. J., Funk, S., *et al.* (2020). Estimating the overdispersion in covid-19 transmission using outbreak sizes outside china. *Wellcome Open Research*, **5**(67), 67.
